# Chromaticity and image processing applied to determine the erosion area of vacuum interrupter contacts

**DOI:** 10.1016/j.heliyon.2025.e42570

**Published:** 2025-02-08

**Authors:** Liting Ma, Zhaoyu Ku, Dongheng Li, Jia Shi, Huajun Dong

**Affiliations:** a School of Mechanical Engineering, Dalian Jiaotong University, Dalian, 116028, China; b School of Traffic and Electrical Engineering, Dalian University of Science and Technology, Dalian, 116052, China

**Keywords:** Vacuum interrupter, Colorimetric, Image processing, Ablation area, Contact

## Abstract

The contact is the core component inside the vacuum switch arc extinguishing chamber. Studying the surface ablation phenomenon of the contact and evaluating the ablation area has an important impact on regulating the evolution of various physical fields in the vacuum arc extinguishing chamber, optimizing the performance of the vacuum arc extinguishing chamber, and extending its service life. This article proposes a method for evaluating the surface ablation area of vacuum switch contacts. This method is based on chromaticity theory and image processing technology. In the RGB, HSI, HSL, and HSV color systems, image feature analysis, pixel statistics, threshold cutting, edge enhancement, and area calculation methods are used to evaluate the ablation area of contact ablation images. Ten sets of test pieces are used for accuracy analysis. The experimental results show that area evaluation can be achieved in the RGB, HSI, HSL, and HSV color systems, and the evaluation accuracy is high in the HSV color system, with an evaluation error controlled within 3 %. This research method can provide evaluation methods and data support for extending the service life of vacuum switches, optimizing contact structures, and other related research.

## 1 Introduction

Vacuum switches utilize the vacuum environment as a medium and have been widely used in aerospace and other medium and low voltage power systems due to their advantages such as compact structure, easy maintenance, no pollution, and strong breaking ability [[1], [2], [3]]. The vacuum arc extinguishing chamber is the main component of vacuum switches, and the failure to break due to factors such as the structure and material of the contacts causing arc reignition is one of the reasons that restricts the rapid development of vacuum switches [[4], [5], [6]].

At present, research on contacts is mainly divided into the following two aspects: (1) Study and analyze the influence of contacts and their structures, materials, etc. on the evolution process of various physical phenomena such as temperature field, electric field, magnetic field, etc. in the vacuum arc extinguishing chamber, providing reference for controlling the arc shape and improving the breaking ability of vacuum switches [7,8]. Among them, scholars such as Li Can [9] proposed a method for directly monitoring the contact temperature of the arc extinguishing chamber. Firstly, a contact model was used to simulate and analyze the electromagnetic temperature multiphysics coupling effect inside the arc extinguishing chamber. Then, partial least squares regression was applied to calculate the environmental temperature, upper contact arm temperature, etc. to achieve real-time monitoring of contact temperature. Scholars such as Dong Huajun [10] used finite element analysis to conduct bidirectional coupling simulation analysis on the temperature field of the arc extinguishing chamber, and obtained the influence of contact structure factors such as contact diameter, contact plate thickness, cup holder thickness, and contact plate slot length on the temperature field. Scholars such as Yang Jinwang [11] studied the magnetic field characteristics between cup shaped longitudinal magnetic contacts under large opening distance conditions, and concluded that increasing the slot rotation angle can effectively enhance the longitudinal magnetic field between contact systems. (2) Research and analyze the physical phenomena on the surface of contact materials, such as ablation, fatigue life, plastic deformation, etc., to optimize the structure of vacuum interrupters and improve their service life [12,13]. Scholars such as Zhang Zaiqin [14] conducted anode melting experiments on four materials, W, Mo, Cr, and Fe, during the high current vacuum breaking process with a fixed cathode of Cu. The highest temperatures of the four materials and the corresponding melting point errors were obtained. At the same time, the scholar also analyzed the surface ablation process of anode materials and summarized the influence of contact materials on anode ablation [15]. Scholars such as Wang Lijun [16] summarized and analyzed the two-dimensional and three-dimensional simulation results of cathode spots on the ablation morphology of contact materials, and discussed the shortcomings of various experimental and simulation studies. Scholars such as Dong Huajun and Li Dongheng have also conducted research on the fatigue life of contacts. Through dynamic simulation analysis, it was found that the convex support plate can make the stress structure distribution of contacts more reasonable [17].

Based on the above analysis, it can be concluded that the research on the contacts, materials, structures, and other related issues of vacuum arc extinguishing chambers is one of the important research directions in this field. However, no scholars have yet evaluated and analyzed the ablation area of the contacts, so there is a lack of quantitative analysis data in this direction. The uneven distribution or excessive ablation area on the surface of the contact material can cause an increase in roughness, and in severe cases, abnormal protrusions and depressions may form on the surface of the contact. Abnormal protrusions and indentations on the surface of the contact can affect the normal emission of electrons by the contact, forming cathodic spots to complete the breaking task, and larger protrusions can prevent the moving and stationary contacts from closing properly. The erosion and cumulative erosion generated on the surface of the contact after a single breaking current or multiple opening and closing actions of the vacuum switch will affect the evolution of various physical fields in the arc extinguishing chamber, thereby affecting the service life of the contact. Therefore, quantitative evaluation of the surface ablation phenomenon of contacts is of great significance for improving the overall performance of vacuum switches.

Image processing technology has the characteristics of non-contact and high accuracy, and has been applied in the research of vacuum switches. Among them, scholars such as Dong Huajun [18] applied image processing technology to evaluate the movement speed of the contacts. Zhang Zuofan [19] applied graphic processing technology to visualize the flow field and temperature field of vacuum arcs. Scholars such as Li Gangsong [20] studied the arc in DC breaking and applied image processing technology to analyze the motion characteristics and study image parameterization. In addition, the combination of color science theory with image processing technology has been carried out for scientific research pages [[21], [22], [23]].Liu Yucheng [24] found that the use of color -based image processing methods can effectively solve the problem of color offset obtained by the collection of collected, making the test results more stable. Hu Yu [25]and other scholars carried out morphological studies on the visible light wave band image of electric halo and discharge, and did some preliminary exploration in terms of color performance.

Based on this, this article proposes a method to quantitatively evaluate the surface ablation area of vacuum switch contacts. This method applies chromaticity theory and image processing related technologies. Firstly, the area is evaluated in four color systems: RGB, HSV, HSL, and HSI. Then, the evaluation accuracy of each color system is analyzed using sample analysis technology. Then, the evaluation plan for the contact ablation area is determined by combining the characteristics of the contact ablation image and the operational characteristics of each color system. Finally, image processing technology is used to improve the evaluation accuracy.

## 2 Evaluation principle and analysis of contact Burnout image features

### 2.1 Evaluation principles

In order to quantify the surface ablation area of the contact, this paper proposes an evaluation method for the surface ablation area of the vacuum interrupter contact. This method uses image processing techniques to evaluate the area in four color dimensions: RGB, HSV, HSL, and HSI. In the RGB color system, each pixel can be represented by three primary colors: red, green, and blue. HSI, HSV, and HSL color systems can all be converted from the RGB color system. HSI is the hue saturation intensity color system, HSL is the hue saturation brightness color system, and HSV is the hue saturation brightness color system [26,27].

The RGB color system is composed of three color components: red (R), green (g) and blue (B). The color of each pixel can be determined by the strength values of these three components. This model is widely used in the color of image processing.

The HSV color system is a color model composed of three components: hue (H), saturation (s) and brightness (V). In the HSV model, hue (H) represents the type of color, saturation (s) represents the purity of the color, and the brightness (v) represents the brightness of the color, which is usually used to more intuitively describe the attributes of the color.

The HSL color system is similar to HSV. The difference is that the brightness (L) component in HSL indicates the degree of light and darkness of the color, which is more suitable to describe the changes in light and surface brightness.

The HSI color system is similar to the HSV system, but it describes the brightness of the color by strength (i), and uses saturation (s) and hue (H) to reflect the purity and type of the color.

**Fig. 1:**
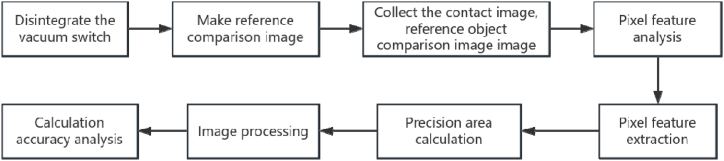
Experimental flow chart.

During the experiment, the vacuum arc extinguishing room after the arc drawing experiment was first disintegrated, and this experiment study was performed according to the process shown in Fig. 1 after disintegration.

**Fig. 2:**
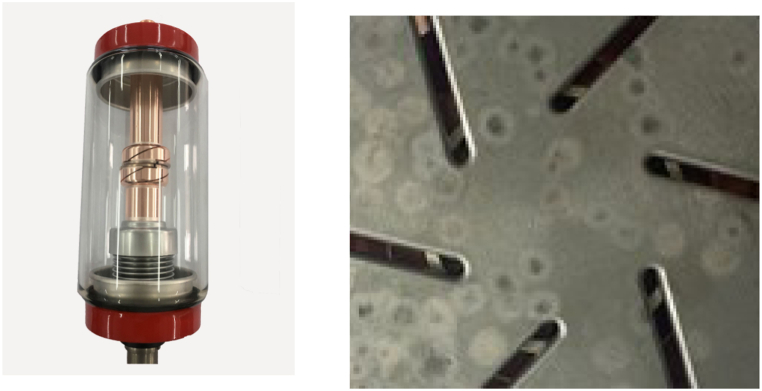
Empty arc extinguishing chamber and contact image.

As shown in Fig. 2, a glass cover vacuum arc extinguishing chamber was selected for this experiment, with a contact diameter of 75 mm. The MotionPro high-speed camera using CMOS cameras has the following main parameters.

(1) Maximum resolution: 1280 x 1024;
(2) Maximum frame resolution: 3 x 105fps;
(3) Data bits: 8 bits, 16 bits, 32 bits;
During the experiment, contact images with burn marks were imported into the Labview virtual instrument platform. Firstly, edge enhancement technology and threshold segmentation method were used to segment the contact area and contact surface burn area. Then, the Vision Builder development package was used to perform pixel statistics on the segmented contact area and contact surface burn area. Namely

(1) $$S=P.\frac{S_{C}}{P_{C}}$$

In the formula: S is the area of the ablation zone, mm^2^; S_C_ is the contact area, mm^2^; P represents the number of pixels in the contact ablation area, while P_C_ represents the number of pixels in the contact area, which can be obtained by counting the number of pixels.

In order to measure the accuracy of this method in evaluating the surface ablation area of the contact, a square specimen with a side length of 1 mm–10 mm was introduced as the specimen. The basic principle of error calculation is similar to the evaluation principle of contact ablation area. The Vision Builder development package is used to perform pixel statistics on the contact area and the test piece area respectively. Namely

(2) $$S_{T}=P_{T}.\frac{S_{C}}{P_{C}}$$

In the formula: S_T_ is the area of the test piece, mm^2^; P_T_ is the number of pixels in the test area, which can be obtained by counting the number of pixels.

After evaluating the area of the test piece using formula (2), the accuracy of this method in area calculation can be obtained by comparing the evaluation results with the actual area of the test piece.

### 2.2 Analysis of image features of contact Burnout

This experiment uses the images that have not been eroded and the contacts that are not eroded and the discount after being cut off 200 times indicates that the images of the erosion are as a research object.

**Fig. 3:**
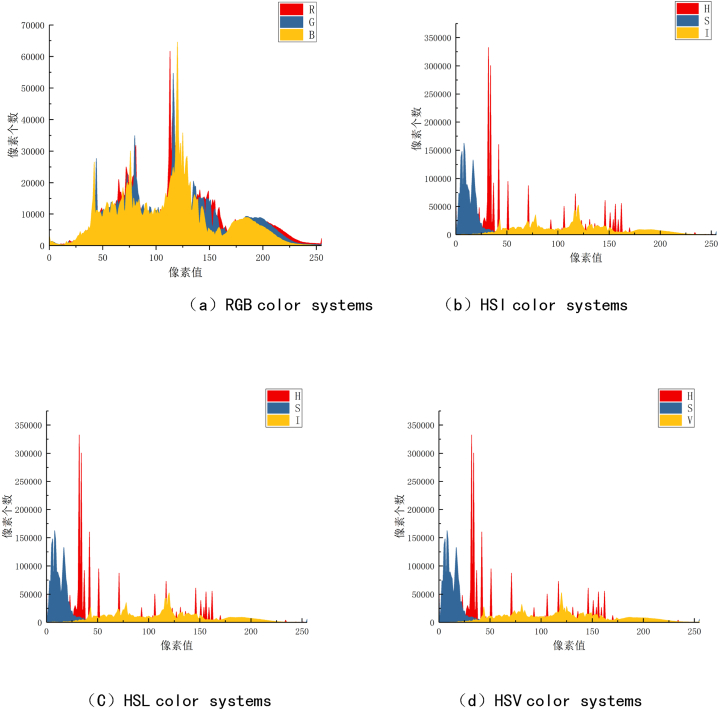
Unproofed image pixel information statistics.

The images that have not been eroded are shown in Fig. 3 (a), and the surface of the contact surface is smooth and no obvious pixel information changes. The ablation area on the surface is similar to circular or oval. The distribution of the obscure areas does not have obvious regularity, but the degree of ablation in the center of the contact is mild. With the increase of the radius, the ablation is increased. In addition, as shown in Fig. 1, the degree of ablation on the left side of the tentacles is higher than the right side of the tentacles. The cause of this phenomenon needs to be further studied and analyzed.

**Fig. 4:**
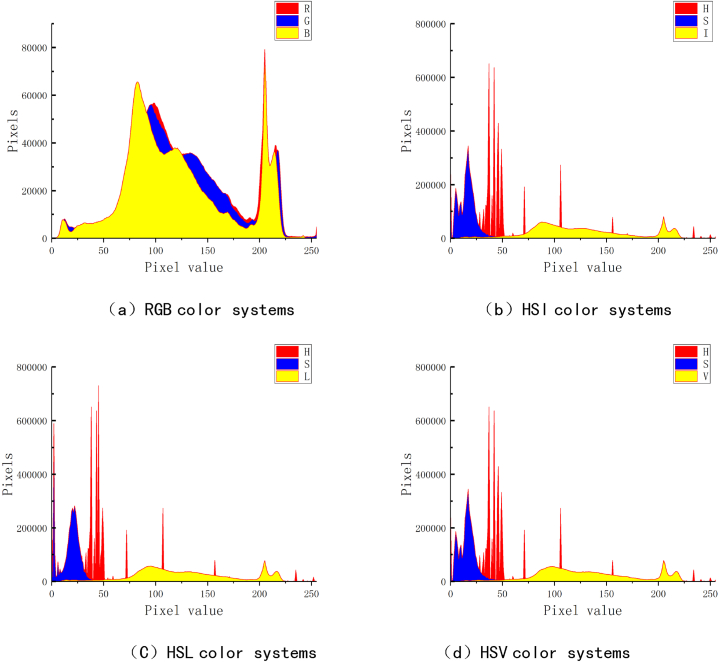
Contact erosion image pixel information statistics.

The contact images and erosion images are introduced into LabView software for pixel value distribution statistical analysis. This experiment is really distributed in the number of pixels of pixel values in the four color systems of RGB, HSV, HSL, and HSI. The statistical results are shown in Fig. 3, Fig. 4.

The changes in the amount of images that have not been eroded in the four color systems are shown in Fig. 3. In the RGB color system, the number of pixels is the peak value of R, G, and B in the three components of R, G, and B, and the number of pixels in the pixel value is 61,632 pixels at the pixel value. The final peak of B component, the number of pixels in the pixel value is 64522 pixels when the pixel value is 120. In the three color systems of HSV, HSL, and HSI, the number of pixels of each component is independently distributed, the overlapping is low, and the distribution of pixels is large. The H component shows a multi -peak form in the three systems. The pixel distribution of S components in the three systems is mainly concentrated in the pixel value of about 0–50. I, V and L's three components are basically the same in the three systems, and they are basically the same, both of which appear between about 75-100 and 200–225. However, the maximum number of pixels of each pixel value of i, V, and L is not more than 20,000.

The contact ablation image contains approximately 4.79 million pixels in total. According to Fig. 4 (a), in the RGB color system, the variation trends of R, G, and B components are basically consistent and can be divided into four regions. When the pixel range of the first region is about 0–50, the number of pixels in the three components steadily increases to around 6000 pixels. The pixel range of the second region is approximately between 51 and 170, and the trend of the three components undergoes the first peak. At this peak stage, the proportion of B component is relatively high, and when the pixel value is 80, the number of pixels is about 65000. The pixel range value of the third region is between 171 and 225, and the trend of the three components shows a second peak. In the second peak stage, the number of pixels in the R, G, and B components all reached about 79000 at a pixel value of about 200, and began to decrease to about 1000 at a pixel value of about 220. In the fourth stage, the pixel range is between 226 and 255, and the number of pixels for the three components remains stable within 1000.

According to Fig. 4(b–d), in the three color systems of HSV, HSL, and HSI, the number of pixels of each component of each component also shows an uniform independent distribution state. The changes in the five components of H, S, I, V, and L The trend of the change in pixel values in the three systems is basically the same as that the change of the change of the images of the contacts is basically the same, but the number of pixels of each weight value will reduce the trend. Among them, the number of pixel values of each pixel value of i, V, and L is reduced, and the maximum value does not exceed 10,000.

The pixel information comparison of the image before and after the contact is found to be found in the RGB system: in the RGB system, when the contact surface is obscured, the red (R) portion may increase, especially when the temperature rises, the ablation area may present red redness. Tune; green and blue (G and B) components may decrease, reflecting the darkening or vagueness of the surface color.

In the HSV system, the ablation area may experience hue changes, and the color of the contact surface changes from metal to red or gray, reflecting the changes in surface temperature and materials. The saturation (s) will be reduced, indicating that the color of the obscure area is no longer pure; the brightness (V) will be reduced, indicating that the brightness of the ablation area will be weakened.

Similar to HSV in the HSL system, the brightness (L) of HSL (L) pays more attention to the light and dark changes of the color. The ablation area may show a darker area, reflecting the increase in the surface roughness of the contamination area of the contact material.

In the HSI system, the strength (i) in HSI changes with the progress of the ablation, and the reduction of the strength indicates that the ability to reflect light on the surface of the contact material weakens.

The pixel distribution of the contact image in the RGB system is more uniform, and the number of three components is more coordinated. In the three color systems of HSV, HSL, and HSI, the number of pixels of each component of images is more independent, which can better reflect the detail information of pixels. , HSI three color systems have strong anti -interference performance in each component.

## 3 Assessment of ablation area of contact image

### 3.1 Assessment of contact crater area

**Fig. 5:**
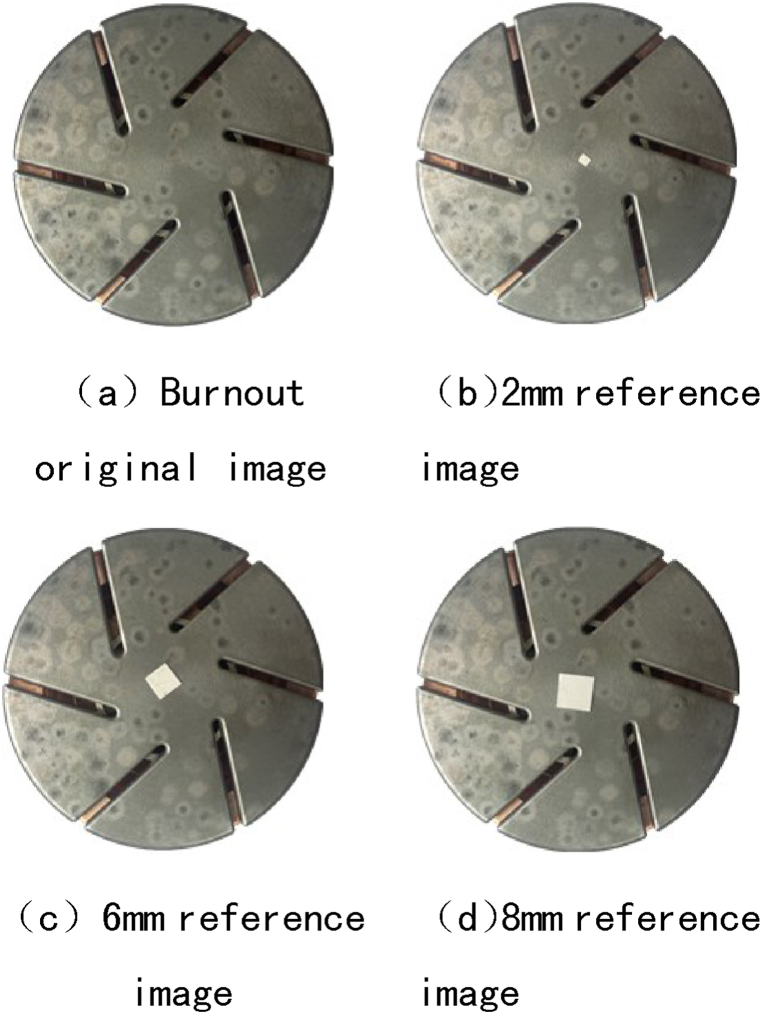
Contact erosion and comparative images.

The specific method for evaluating the area of contact melting pits is as follows: (1) background segmentation is performed on the ablated contact image, and standard square test pieces with a side length of 1–10 mm are made (rounded); (2) Collect and transmit contact ablation images and comparative images, with partial acquisition results shown in Fig. 5; (3) Based on four color systems of RGB, HSI, HSL, and HSV, threshold segmentation is performed to select the contact area, ablation area, and specimen area; (4) Using a combination of filtering and integration functions to eliminate image noise and enhance edge information in various regions; (5) Using pixel statistics to evaluate the number of pixels in different regions; (6) Use formula 1 to evaluate the area of contact erosion, and use formula 2 to calculate the evaluation accuracy.

#### 3.1.1 Evaluation and error analysis of ablation area in RGB color system

In the RGB color system, the color characteristics of each pixel in a color image can be represented by three primary colors and quantity values, and the relationship between the four is shown in Equation (3):

(3) C[C]=R[R]+G[G]+B[B]

In the formula, C, R, G, and B represent the quantity values of mixed colors and three primary colors; [C] [R], [G], [B] are units of mixed colors and three primary colors.

The contact ablation image contains a large number of pixels, and the chromaticity characteristics of each pixel are determined by the different component values of the R, G, and B primary colors. Through experimental statistical analysis, it has been found that the area of the ablation area and specimen area in the contact ablation image is directly related to the size of the R component in the RGB ratio [28]. Part of the statistical results are shown in Table 1. When the areas are the same, the standard deviation of the R component is the smallest. When the area is different, the larger the ablation area and specimen area, the smaller the standard deviation of the R component value, and the more stable the data statistics are [29]. Therefore, this article chooses the method of extracting the R component to achieve the effect of image grayscale and proceed with the next step of operation.

**Table 1:** Standard deviation of RGB values for different components.

Standard deviation of each component	4 mm^2^	9 mm^2^	16 mm^2^	25 mm^2^
R	50.01	49.97	49.84	49.62
G	50.88	52.25	50.77	51.40
B	52.91	53.36	52.94	53.43

**Fig. 6:**
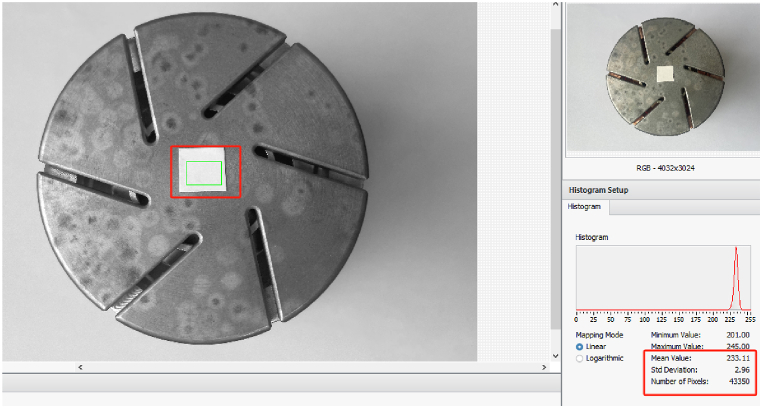
Reference object threshold extraction.

After completing the gray of the contact image and the comparison image image, threshold segmentation is performed for the contact area, the ablation area and the test area area. Through the LabView platform Vision Builder processing package, the target image amplification value is 100 and only for pixel feature statistics for the target area. Fig. 6 is a pixel information statistics image with a trial area with a side length of 10 mm.

After pixel analysis, the pixel range value of the contact area is determined by (180, 220), the area value of the area pixels in the ablation area is between (70, 170), and the area of the pixel area of the test film is between (220, 240). Through Formula 1 and Formula 2, the area area and test area are evaluated, as shown in Table 2.

**Table 2:** Area calculation results and errors.

*Image*	*Ablation image*	*Test piece edge length 1 mm*	*Test piece edge length 2 mm*	*Test piece edge length 3 mm*	*Test piece edge length 4 mm*	*Test piece edge length 5 mm*
Area/mm^2^	149.78	1.01	4.14	9.21	16.64	25.64
Error	–	1 %	3.5 %	2.33 %	4 %	2.56 %

*Image*	*Test piece edge length 6 mm*	*Test piece edge length 7 mm*	*Test piece edge length 8 mm*	*Test piece edge length 9 mm*	*Test piece edge length 10 mm*
Area/mm^2^	37.14	50.42	66.26	85.01	105.32
Error	3.17 %	2.89 %	3.53 %	4.95 %	5.32 %

The evaluation results indicate that when evaluating the total area of the ablation area using this method, the larger the specimen area, the greater the evaluation error. When the side length of the test piece area is 10 mm, the evaluation error reaches 5.23 %, which may be caused by image noise. When performing threshold segmentation, the larger the area of the test piece, the larger its pixel range, and the more pixels there are, resulting in a decrease in the accuracy of the evaluation results. In order to improve the accuracy of evaluation, it is considered to introduce image processing techniques to reduce the impact of image noise on the evaluation results.

#### 3.1.2 Evaluation and error analysis of ablation area in HSI, HSV, and HSL color systems

Convert RGB system into three color systems: HSI, HSV, and HSL [30]. The formulas for converting RGB models into HSI, HSV, and HSL models are as follows:

$$\begin{array}{l} I=\frac{1}{3}.\sum \left(R+G+B\right)\\ V=C_{\max}\\ L=\frac{C_{\max}+C_{\min}}{2}\\ H=\left\{ \begin{array}{l} 0\;\left(\text{if}\;\text{Cmax}=\text{Cmin}\right)\\ \left(\frac{G-B}{\Delta }\right)\times 60{}^{\circ}\left(\text{if}\;\text{Cmax}=R\right)\\ \left(\frac{B-R}{\Delta }+2\right)\times 60{}^{\circ}\left(\text{if}\;\text{Cmax}=G\right)\\ \left(\frac{R-G}{\Delta }+4\right)\times 60{}^{\circ}\left(\text{if}\;\text{Cmax}=B\right) \end{array}\right.\\ S=\left\{ \begin{array}{l} 0\left(\text{if}\;\text{Cmax}=\text{Cmin}\right)\\ \Delta \left(\text{if}\;\text{else}\right) \end{array}\right. \end{array}$$

In the formula:*C*_max_*=*max*(R,G,B)*;

*C*_min_*=*min*(R,G,B)*;

Δ = Cmax-Cmin.In each color system mentioned above, the three components exist independently of each other. Among them, the H and S components determine the color information of pixels. H determines the color attribute of pixels, and S determines the color purity of pixels. The size of H depends on the angle between the color attribute of the pixel and the reference color (red), with a phase angle of 120° between the three primary colors. This indicates that H red = 0° or 360°, H green = 120°, and H blue = 240°. S is determined by the distance between the pixel and the intersection of the color plane and the brightness axis.

**Fig. 7:**
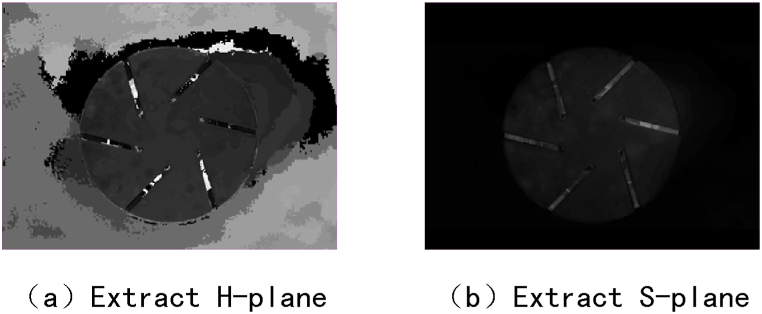
Extract H and S plane processing results.

Perform grayscale processing on the comparison images of the contact ablation image set in three color systems: HSI, HSV, and HSL. The experimental results show that if H or S planes are extracted from the above three color systems, all image information cannot be displayed. The experimental results are shown in Fig. 7. Therefore, the HSI, HSV, and HSL color systems can perform image grayscale and threshold segmentation at the I, V, and L levels respectively.

Using Labview pixel statistics, the pixel ranges of the contact area, ablation area, and specimen area within the three color systems mentioned above are shown in Table 3. Using (1), (2) again, evaluate the contact ablation area and specimen area. Some evaluation results are shown in Table 4.

**Table 3:** Pixel range of target area in different color spaces.

Region	Contact region	Ablation region	Test region
HSI	(175,210)	(65,170)	(223,238)
HSV	(168,207)	(72,168)	(210,230)
HSL	(170,200)	(68,160)	(215,233)

**Table 4:** Pixel range of target area in different color spaces.

Image	*Ablation image*	*Test piece edge length 1 mm*	*Test piece edge length 3 mm*	*Test piece edge length 5 mm*	*Test piece edge length 7 mm*	*Test piece edge length 9 mm*
HSI	167.6	1.01	9.87	26.73	52.24	83.79
HSV	152.24	1.01	9.14	25.71	50.37	83.96
HSL	131.97	0.97	8.42	19.58	40.19	52.93
HIS Error	–	1 %	9.67 %	6.92 %	6.61 %	3.44 %
HSV Error	–	1 %	1.55 %	2.84 %	2.79 %	3.65 %
HSL Error	–	3 %	6.44 %	21.68 %	17.98 %	34.65 %

The total area of contact ablation was evaluated in the RGB, HSV, HSL, and HSI color systems as 149.78 mm^2^, 152.24 mm^2^, 131.97 mm^2^, and 167.6 mm^2^, respectively. The maximum ablation area of the melt pit in each color system was below 20 mm^2^.

### 3.2 Comparison of evaluation results

**Fig. 8:**
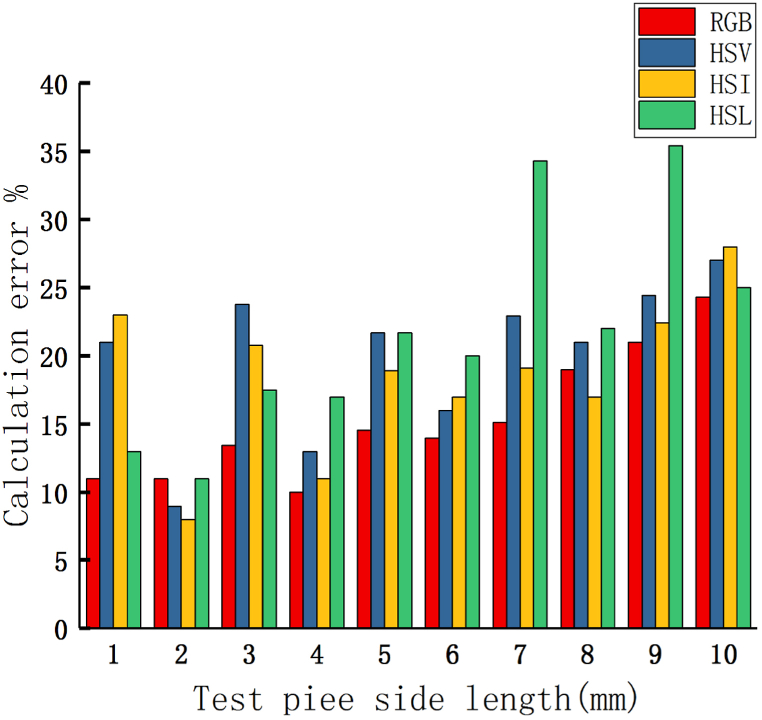
Comparison of calculation errors.

In summary, the evaluation method for the area of contact ablation proposed in this article can be achieved in four color systems: RGB, HSV, HSL, and HSI, but the evaluation accuracy varies for each space. The comparison chart of the evaluation accuracy of four color systems in evaluating different areas is shown in Fig. 8.

(1) There is a significant evaluation error in the HSL and HSI color systems. When calculating the area of a test piece with an area of 81mm_2_ in the HSL color system, the maximum evaluation error is 34.65 %. The maximum measurement error for measuring the area of 9 mm^2^ test pieces in the HIS color system is about 10 %. Based on the characteristics of the contact ablation image, it is found that the shape of a single ablation area of the contact is approximately circular, and the radius of the ablation area does not exceed 3 mm. When evaluating the ablation area of the contact, in addition to considering the stability of the area calculation results, the calculation accuracy of small area specimens should also be emphasized. Therefore, the calculation errors of the above two color systems are relatively large and are not suitable for evaluating the contact ablation area.
(2) In RGB and HSV color systems, the measurement accuracy of the test piece area is relatively high, and the measurement error can be controlled below 3.5 % when measuring the test piece area below 9 mm^2^。

In the RGB color system, the features of each pixel need to be described by three components together. The RGB color system is a color space defined based on colors that can be recognized by the human eye. It is the superposition effect of three primary colors and cannot clearly express the detailed features of each pixel. In the HSV color system, it is possible to balance the pixel information of each pixel and describe the detailed information such as chromaticity, saturation, and brightness of each pixel. The calculation model of the HSV color system is approximately a six cone, and through the conversion formula mentioned earlier, it can be seen that the V (brightness) component takes the maximum value of each component of the RGB color system. According to Fig. 4 (a), it can be seen that the B component of the contact ablation image has the highest value in the RGB color system. The B component can be used to measure the brightness of the image in the system. In cases where the color of the contact image is not bright, the pixel information caused by the brightness when evaluating the ablation area is one of the main reference indicators for determining the pixel range of the ablation area. And through the evaluation results, it was found that the average error of the RGB system in evaluating each area is 2.88 %. The average error of the HSV system in evaluating each area is 2.64 %.

Based on the characteristics of the contact ablation image, the evaluation results of each color system, and the operational characteristics of each color system, it was found that the HSV color system can fully describe the characteristics of each pixel in the ablation image. Moreover, when evaluating the area of each test piece, the stability and accuracy of the evaluation results are relatively high. Therefore, this method uses the HSV color system for evaluation, and on this basis, image processing is carried out to improve the accuracy of the evaluation.

## 4 Image processing and analysis of calculation results

### 4.1 Image processing

To further improve the accuracy of the calculation results, this article uses four image denoising methods provided by the Labview virtual instrument platform: low-pass filtering, Gaussian filtering, median filtering, bilateral filtering, and five image enhancement processing methods: Sobel, Laplacian, Prewitt, Roberts, and Difference. Among them, Prewitt, Roberts, and Differentiation, three image enhancement methods, were unable to fully preserve the effective information of the image during image processing. Therefore, eight experimental schemes for image processing were preliminarily determined, as shown in Table 5.

**Table 5:** Image processing scheme.

Experimental plan	Image denoising methods	Image enhancement methods
1	Low pass filtering	Sobel
2	Gaussian filtering	Sobel
3	Median filtering	Sobel
4	Bilateral filtering	Sobel
5	Low pass filtering	Laplacian
6	Gaussian filtering	Laplacian
7	Median filtering	Laplacian
8	Bilateral filtering	Laplacian卷积

During the experiment, the filtering kernels of the four filtering functions were set to 3, and the convolution kernels of Sobel and Laplacian convolutions were set to 7. The experiment found that using Gaussian filtering for denoising and Laplacian convolution function for image enhancement resulted in the highest computational accuracy. After image processing, threshold segmentation is performed again to determine that the pixel range value of the contact area is (175–220), the pixel range value of the ablation area is (73–162), and the pixel range value of the reference area is between (220–234). The calculation results are shown in Table 6.

**Table 6:** Area calculation results and errors after image processing.

Image	*Ablation image*	*Test piece edge length 1 mm*	*Test piece edge length 2 mm*	*Test piece edge length 3 mm*	*Test piece edge length 4 mm*	*Test piece edge length 5 mm*
Area/mm^2^	151.17	1	4.11	9.07	16.23	25.99
Error	–	0 %	2.75 %	0.78 %	1.43 %	3.96 %

Image	*Test piece edge length 6 mm*	*Test piece edge length 7 mm*	*Test piece edge length 8 mm*	*Test piece edge length 9 mm*	*Test piece edge length 10 mm*
Area/mm^2^	37.16	50.19	66.18	82.69	104.41
Error	3.22 %	2.43 %	3.41 %	2.09 %	4.41 %

### 4.2 Analysis of calculation results

After image denoising and image enhancement processing in the HSV color model, the evaluation error of this method was reduced. The total ablation area of the contact ablation image is 151.17 mm^2^, with the maximum ablation area being 10.37 mm^2^. Compared with the results before image processing, the average evaluation error for each area interval is about 2.45 %; When calculating the area range within 16 mm^2^, the evaluation error can be controlled within 3 %.

**Fig. 9:**
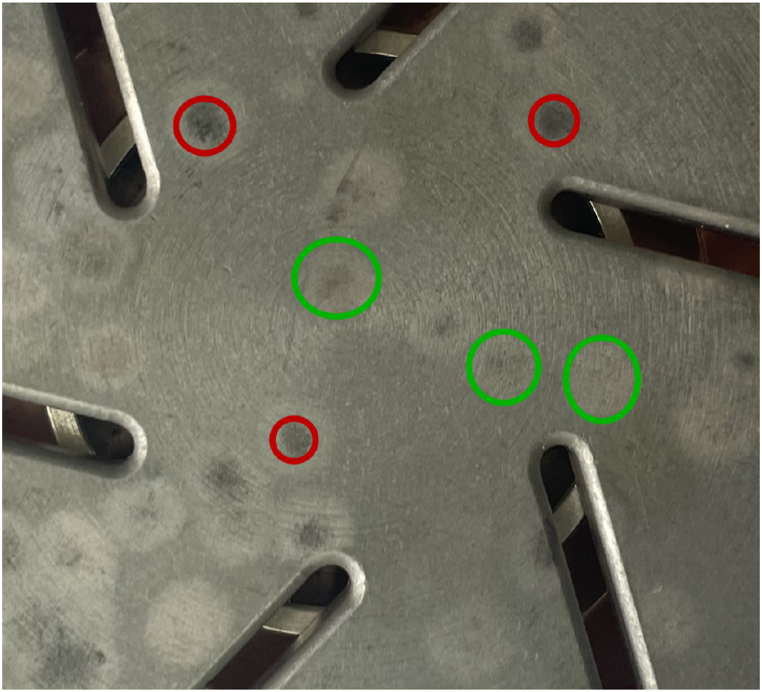
Contact surface erosion phenomenon.

During the experiment, it was found that there are several factors that affect the calculation accuracy of this algorithm: (1) The contact surface selected for this experiment is uniformly distributed with six slots, which are used to reduce the erosion of the contact surface and regulate the electric field, magnetic field, and temperature field during the opening and closing process. As shown in Fig. 2, the pixel values in this area are close to those in the contact ablation area. During pixel count counting, some pixels in the slotted area were mistakenly counted in the ablation area, resulting in the calculation result being larger than the actual area. (2) According to Fig. 9, the burnt area on the surface of the contact can be divided into two categories. One type of red area has a lighter degree of erosion, with a pixel range of around (101–151), while the other type of green area has a heavier degree of erosion, with a pixel range of around (70–170). When calculating the ablation area, the two types of areas were not separately counted, and only the average pixel range of areas with higher ablation degree was used as a threshold parameter for pixel statistics, resulting in calculation results that were inconsistent with reality.

## 5 Conclusion

This article focuses on the evaluation of the surface ablation area of vacuum switch contacts, and proposes an evaluation method for the surface ablation area of vacuum switch contacts based on colorimetric research theory and image processing technology. This method is based on four color systems: RGB, HSV, HSL, and HSI. Firstly, pixel statistical analysis technology is used to analyze the characteristics of contact ablation images; Then, using techniques such as edge enhancement and threshold segmentation, the pixel information of the ablation area is cut and counted to achieve area evaluation; Reuse test pieces for error analysis;

Secondly, by comparing the image characteristics before and after contacts are eroded by image feature analysis technology, it is found that in the four color systems of RGB, HSV, HSL, and HSI, the changes in each component can reflect some physical changes during the contacts being ablated. In the future, problems such as morphological changes and mass loss of the contact material surface can be further studied based on the physical characteristics of different components;

Finally, image processing technology is applied to improve the accuracy of ablation area evaluation. Evaluating the contact ablation area in the HSV system has high reference value. After image denoising and image enhancement processing in the HSV color system, the evaluation error can be reduced by 0.19 %.The results of this research can provide technical support for subsequent use of the theoretical theory of color science to evaluate the arc recovery intensity of the medium recovery intensity on the surface of the tentacle material.

## CRediT authorship contribution statement

**Liting Ma:** Writing – review & editing, Writing – original draft, Software, Formal analysis, Data curation, Conceptualization. **Zhaoyu Ku:** Software. **Dongheng Li:** Software, Resources. **Jia Shi:** Software. **Huajun Dong:** Funding acquisition, Formal analysis.

## Declaration of competing interest

The authors declare the following financial interests/personal relationships which may be considered as potential competing interests: DongHuajun reports financial support, administrative support, article publishing charges, and statistical analysis were provided by 10.13039/501100001809National Natural Science Foundation of China. If there are other authors, they declare that they have no known competing financial interests or personal relationships that could have appeared to influence the work reported in this paper.
